# The HECT domain ubiquitin ligase HUWE1 targets unassembled soluble proteins for degradation

**DOI:** 10.1038/celldisc.2016.40

**Published:** 2016-11-08

**Authors:** Yue Xu, D Eric Anderson, Yihong Ye

**Affiliations:** 1 Laboratory of Molecular Biology, National Institute of Diabetes and Digestive and Kidney Diseases, National Institutes of Health, Bethesda, MD, USA; 2 Advanced Mass Spectrometry Core Facility, National Institute of Diabetes and Digestive and Kidney Diseases, National Institutes of Health, Bethesda, MD, USA

**Keywords:** HUWE1, p97/Cdc48, protein quality control (PQC), ubiquitin, unassembled soluble protein degradation (USPD)

## Abstract

In eukaryotes, many proteins function in multi-subunit complexes that require proper assembly. To maintain complex stoichiometry, cells use the endoplasmic reticulum-associated degradation system to degrade unassembled membrane subunits, but how unassembled soluble proteins are eliminated is undefined. Here we show that degradation of unassembled soluble proteins (referred to as unassembled soluble protein degradation, USPD) requires the ubiquitin selective chaperone p97, its co-factor nuclear protein localization protein 4 (Npl4), and the proteasome. At the ubiquitin ligase level, the previously identified protein quality control ligase UBR1 (ubiquitin protein ligase E3 component n-recognin 1) and the related enzymes only process a subset of unassembled soluble proteins. We identify the homologous to the E6-AP carboxyl terminus (homologous to the E6-AP carboxyl terminus) domain-containing protein HUWE1 as a ubiquitin ligase for substrates bearing unshielded, hydrophobic segments. We used a stable isotope labeling with amino acids-based proteomic approach to identify endogenous HUWE1 substrates. Interestingly, many HUWE1 substrates form multi-protein complexes that function in the nucleus although HUWE1 itself is cytoplasmically localized. Inhibition of nuclear entry enhances HUWE1-mediated ubiquitination and degradation, suggesting that USPD occurs primarily in the cytoplasm. Altogether, these findings establish a new branch of the cytosolic protein quality control network, which removes surplus polypeptides to control protein homeostasis and nuclear complex assembly.

## Introduction

Many eukaryotic proteins function in multi-subunit complexes with a stoichiometry that needs to be strictly maintained. It is thought that the degradation of unassembled subunits might be an essential mechanism that controls complex stoichiometry [1, 2]. Elimination of unassembled proteins is also crucial for protein homeostasis because unassembled proteins often contain exposed hydrophobic segments that can lead to protein aggregation and cytotoxicity. In fact, a major effort in developing anti-cancer therapies that target proteostasis-addicted tumors is based on the assumption that unbalanced protein assembly due to aneuploidy or other genome instabilities in cancer cells render them more susceptible to chemicals that disturb the proteostasis network [3]. In this regard, identification of cellular components mediating the degradation of unassembled proteins may reveal novel anti-cancer targets.

Membrane and secreted protein complexes are usually assembled in the endoplasmic reticulum (ER) after individual subunits have been imported into the ER [4]. The assembly process is subject to a strict checkpoint regulation enforced by an efficient protein quality control (PQC) mechanism. The ER PQC pathway employs chaperones, lectins and other enzymes to monitor the assembly process, identifying unassembled polypeptides for retrotranslocation, ubiquitination and proteasomal degradation in the cytosol [5, 6]. This conserved process is termed ER-associated protein degradation (ERAD) [7], which is critical for cell viability because unassembled polypeptides can interfere with normal protein assembly when they become misfolded or form non-specific interactions. Unassembled ER proteins can also co-aggregate with essential cellular factors such as chaperones to cause ER stress, which if not rectified, can lead to cell death [8].

Many proteins in the cytosol and nucleus also form multi-subunit assemblies, but the mechanism by which cells eliminate unassembled soluble proteins is not well understood. Several studies have investigated the mechanism of cytoplasmic and nuclear PQC, which remove misfolded or damaged proteins from the cytoplasm and nucleus, respectively [9–15]. These studies identified several pathways that target misfolded proteins of different classes to the proteasome for degradation. For example, the ribosome-associated ubiquitin ligase Ltn1 in budding yeast recognizes and ubiquitinates defective translation products due to non-stop messenger RNAs [12, 13]. In mammalian cells, a chaperone-associated ubiquitin ligase named RNF126 targets mislocalized membrane proteins for degradation [15]. However, these studies did not use substrates representing unassembled polypeptides. Therefore, it is unclear whether these cytosolic PQC pathways play a role in unassembled soluble protein degradation (USPD).

To date, the best-characterized cytosolic quality control pathway is the N-end rule pathway, which mediates the degradation of substrates bearing an N-terminal destabilizing element termed ‘degron’. The N-end rule substrates have been classified into three major groups: those with an N-terminal destabilizing residue, those with an exposed acetylated N-terminal methionine residue and a group of proteins with an N-terminal initiator methionine followed by a hydrophobic residue [16]. A major ubiquitin ligase responsible for degradation of non-acetylated N-end rule substrates is UBR1 and the related enzymes UBR2 and UBR3. In yeast, a protein named CNOT4 was recently identified as the ubiquitin ligase for an unassembled soluble protein carrying an exposed acetylated N-terminal methionine [2]. It is conceivable that some USPD substrates may carry one of the above-mentioned ‘degrons’, but for those without a predicted N-end rule ‘degron’, how they are targeted for degradation is unclear.

Here, we established model substrates to study N-end rule independent USPD in mammalian cells. Our study establishes HUWE1 as an enzyme that ubiquitinates substrates bearing exposed hydrophobic residues due to incomplete assembly to cause their degradation by the proteasome. We identify endogenous HUWE1 substrates, which reveal a new surveillance system that safeguards the proteostasis network of the eukaryotic cells.

## Results

### Unassembled soluble proteins are degraded by the proteasome

To study the mechanism of USPD, we sought proteins that function in small complexes consisting of two or three subunits with well-defined interaction interface because structural information could potentially reveal the cause of their instability on disassembly. The farnesyl transferase complex fits these criteria as it contains two subunits, protein farnesyltransferase alpha (FNTA) and protein farnesyltransferase beta (FNTB), which catalyze the transfer of farnesyl moiety from farnesyl diphosphate to a cysteine at the C-terminus of a CAAX sequence [17]. Structural studies showed that the interface between FNTA and FNTB is composed of both non-polar and polar residues [18]. FNTA has an alanine residue next to the initiator methionine, raising the possibility that unassembled FNTA might be degraded by the UBR1-mediated cytosolic PQC pathway [16, 19].

**Figure 1:**
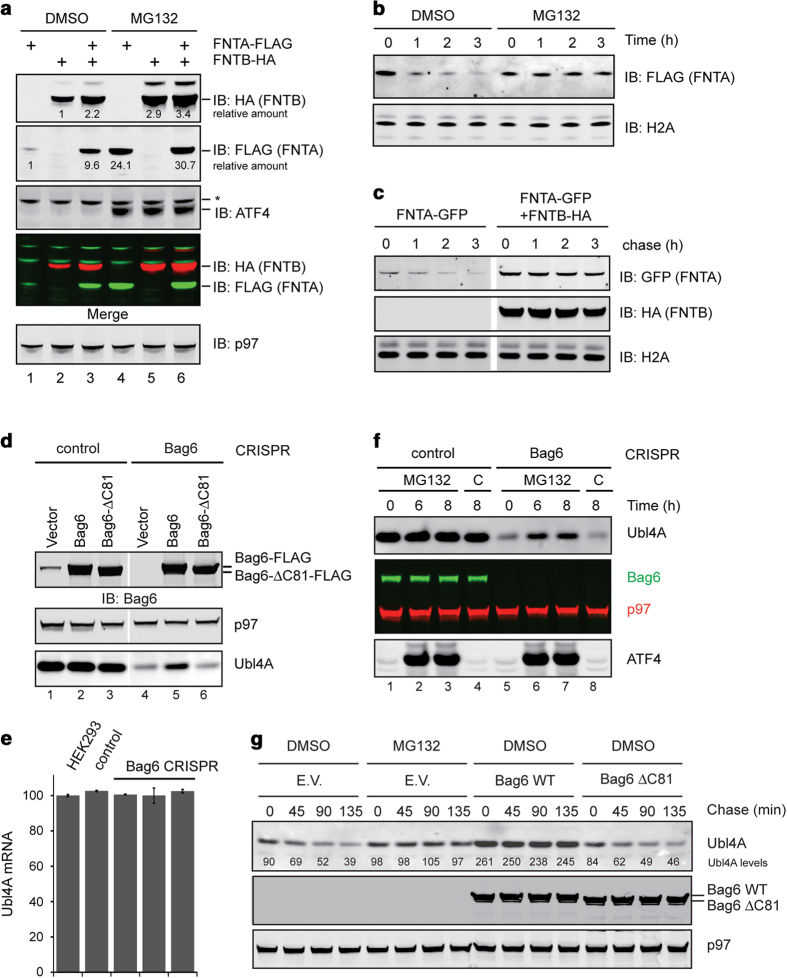
Degradation of unassembled soluble proteins by the proteasome. (**a**–**c**) Unassembled FNTA is degraded by the proteasome. (**a**) Cells transfected with FNTA-FLAG and FNTB-HA either individually or in combination were treated with dimethyl sulfoxide (DMSO) as a control or MG132 (20 μm, 6 h). Whole-cell extracts were analyzed by immunoblotting (IB). Asterisk, non-specific band. (**b**) Cycloheximide chase analysis of FNTA degradation in HEK293 T cells transfected with FNTA-FLAG. Where indicated, cells were treated with MG132 (20 μm). (**c**) Cycloheximide chase analysis of FNTA degradation in HEK293T cells transfected with FNTA-FLAG alone or together with FNTB-HA. (**d**–**g**) Unassembled Ubl4A is regulated by proteasomal degradation. (**d**) Whole-cell extracts from control or Bag6 null CRISPR cells were analyzed by immunoblotting. (**e**) The Ubl4A messenger RNA (mRNA) level in parental 293T cells, control and Bag6 null clones was analyzed by qRT-PCR. Error bars, s.e.m. (*n*=3). (**f**) Whole-cell extracts from cells treated as indicated (MG132, 20 μm 6 h; C, chloroquine 50 μM) were analyzed by immunoblotting. (**g**) Bag6 CRISPR cells transfected as indicated were treated with cycloheximide in the presence of either DMSO as a control or MG132 (20 μm). Cell lysates prepared at the indicated time points were analyzed by immunoblotting. EV, empty vector.

We generated constructs over-expressing FLAG-tagged FNTA and hemagglutinin (HA)-tagged FNTB, respectively. These proteins were ectopically expressed at a much higher level than their endogenous partners, and thus were mostly in an unassembled state. When expressed individually, FNTB was readily detected by immunoblotting (Figure 1a, lane 2), but FNTA could only be expressed at a low level (lane 1). On co-expression, the level of FNTB was modestly upregulated (lane 3 vs lane 2), whereas the FNTA level was dramatically increased (lane 3 vs lane 1). Moreover, treatment with the proteasome inhibitor MG132 also increased the level of FNTA and FNTB (lanes 4–6). These results indicate that both unassembled FNTA and FNTB are USPD substrates degraded by the proteasome, but FNTA seems to have a much shorter half-life than FNTB. Indeed, a translation shut-off experiment confirmed that ectopically expressed FNTA was unstable, but it could be stabilized by MG132 treatment (Figure 1b) or by co-expression of FNTB (Figure 1c).

We next tested whether USPD is applicable to other soluble protein complexes, particularly under endogenous expression conditions. We chose the multi-functional Bag6 chaperone holdase complex because it only has 3 subunits, Bag6, Ubl4A and Trc35, and because the interactions between Bag6 and its partners have been well characterized [20]. Moreover, unlike FNTA, Ubl4A has a charged residue next to the initiator methionine. Thus, if unassembled Ubl4A is also a USPD substrate, characterization of its degradation should shed new insights on USPD.

To study whether the stability of endogenous Ubl4A is dependent on assembly with Bag6, we used the CRISPR technology to generate HEK293T cells deficient in Bag6. Immunoblotting showed that the steady state level of endogenous Ubl4A was significantly reduced in Bag6 null cells (Figure 1d, lane 4 vs 1). Transient expression of FLAG-tagged wild-type (WT) Bag6, but not of a mutant lacking the C-terminal Ubl4A-binding domain (Bag6ΔC81) partially restored the level of Ubl4A (lanes 5 vs 6), suggesting that down-regulation of Ubl4A is due to lack of assembly with Bag6. qRT-PCR analysis showed that the Ubl4A messenger RNA level was comparable between control and Bag6 null cells (Figure 1e), whereas a radiolabeling experiment showed a similar rate of Ubl4A protein synthesis in control and Bag6 null cells during a short labeling period (Supplementary Figure S1A). These results suggest a post-translational mechanism that down-regulates endogenous Ubl4A in Bag6 deficient cells.

Unassembled Ubl4A is degraded by the ubiquitin proteasome system because exposing Bag6 null cells to MG132 but not to the lysosomal inhibitor chloroquine increased Ubl4A (Figure 1f, lanes 6 and 7 vs 5 and 8). A translation shut-off assay further confirmed that in Bag6 null cells, endogenous Ubl4A was short-lived, and its turnover could be blocked by MG132 or by expression of WT Bag6 but not the Bag6ΔC81 mutant (Figure 1g). Moreover, Ubl4A-FLAG immunoprecipitated from Bag6 null cells under denaturing conditions contained ubiquitin conjugates, but the level of ubiquitinated Ubl4A was reduced upon re-expression of Bag6 (Supplementary Figure S1B). Finally, on overexpression, Ubl4A bearing a green fluorescent protein (GFP) tag was also downregulated in Bag6 null cells, but it accumulated to a higher level upon MG132 treatment (Supplementary Figure S1C). Collectively, these results suggest that like membrane proteins, lack of assembly can generally lead to degradation of unassembled soluble proteins by the proteasome.

### p97 and Npl4 are required for USPD

**Figure 2:**
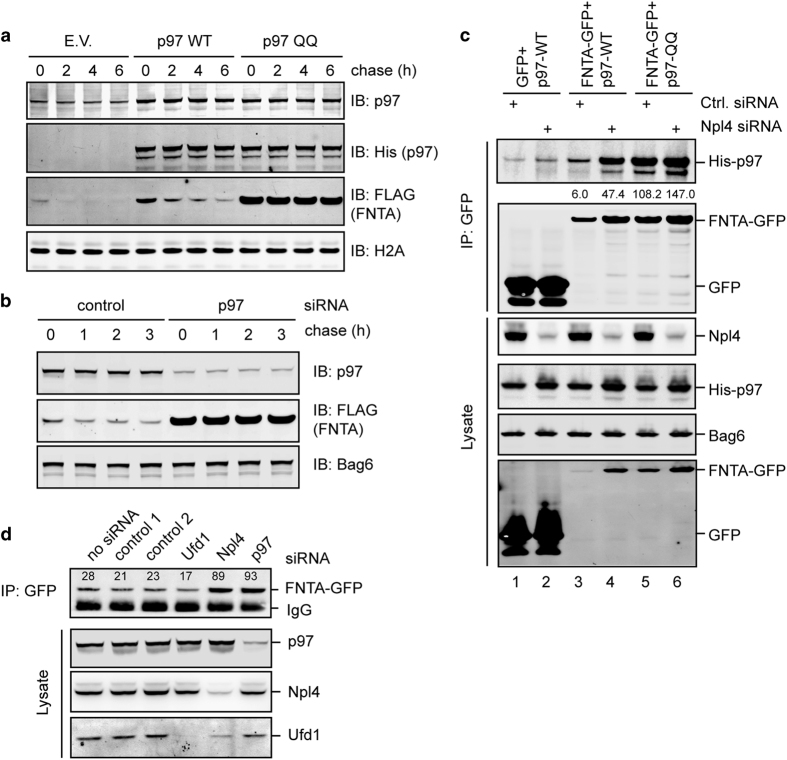
Degradation of unassembled soluble proteins requires p97 and Npl4. (**a**) A p97 dominant negative mutant inhibits the degradation of unassembled FNTA. HEK293T cells transfected with FNTA-FLAG together with the indicated plasmids were subject to cycloheximide chase analysis. (**b**) Knockdown of p97 stabilized FNTA. HEK293T cells transfected with FNTA-FLAG together with the indicated siRNA were analyzed by cycloheximide chase. (**c**) Interaction of p97 with FNTA. Cells transfected with the indicated plasmids and siRNAs were subject to immunoprecipitation and immunoblotting analysis. (**d**) Knockdown of Npl4 but not Ufd1 stabilizes FNTA. Cells stably expressing FNTA-GFP were transfected with the indicated siRNAs. The level of FNTA-GFP was measured by immunoblotting analysis of immunoprecipitated samples. A fraction of whole-cell extracts were directly analyzed to verify knockdown efficiency.

As the p97-Ufd1-Npl4 complex is a conserved ATPase complex widely implicated in degradation of misfolded proteins including unassembled membrane proteins and a cytosolic protein subunit [1, 21], we tested whether it plays a role in USPD. First, we expressed FNTA together with a His-tagged p97 mutant defective in ATP hydrolysis (QQ) because it has a well-established dominant negative effect on p97-dependent processes [22–24]. As a control, we expressed WT p97. A translation shut-off experiment showed that p97 QQ but not WT p97 almost completely blocked FNTA degradation (Figure 2a). Inhibition of p97 ATPase activity by p97 QQ or a specific chemical inhibitor NMS-873 [25] also significantly stabilized overexpressed Ubl4A-GFP (Supplementary Figure S2A and B), suggesting that ATP hydrolysis by p97 might be required for degradation of unassembled soluble proteins.

To further confirm the involvement of p97 in USPD, we used small interfering RNA (siRNA) to deplete endogenous p97, which resulted in a similar stabilization of overexpressed FNTA-FLAG (Figure 2b). Furthermore, co-immunoprecipitation showed that unassembled FNTA interacted with both WT p97 and the QQ mutant, but the p97 QQ mutant associated with FNTA much more strongly than WT p97 (Figure 2c, lanes 5 vs 3). This is consistent with previous reports that the ATPase defective p97 mutant can function as a substrate trap due to a defect in substrate release. These results further suggest a direct role of p97 in USPD. Because expression of p97 QQ did not reduce ubiquitinated Ubl4A (Supplementary Figure S2C), p97 appears to act downstream of ubiquitination.

Because Npl4 and Ufd1 act together to assist p97 in ERAD [22, 26, 27], we tested whether these factors are involved in USPD. Interestingly, knockdown of Npl4, but not Ufd1, reproducibly increased the steady state level of FNTA-GFP and Ubl4A (Figure 2d, Supplementary Figure S2D). These results suggest that Npl4 is involved in USPD. Immunoprecipitation of FNTA from control and Npl4-depleted cells showed that the interaction between p97 and FNTA is not dependent on Npl4 (Figure 2c), suggesting that Npl4 acts downstream of substrate recruitment to facilitate USPD.

### Characterization of the destabilizing element in unassembled Ubl4A

As Ubl4A does not have a hydrophobic residue next to the initiator methionine, its degradation might not involve the N-end rule pathway. Indeed, siRNA-mediated knockdown of UBR1 did not affect the Ubl4A level (Supplementary Figure S3A), although a similar treatment stabilized FNTA (Supplementary Figure S3B). Co-depletion of UBR2 and UBR3 together with UBR1 led to a further increase in FNTA (Supplementary Figure S3B), suggesting that these ligases act in parallel to degrade FNTA. Knockdown of RNF126, a ubiquitin ligase involved in degradation of mislocalized membrane proteins [15] also had no effect on Ubl4A (Supplementary Figure S3A), neither did knockdown of CNOT4 (Supplementary Figure S3C). These results suggest that Ubl4A represents USPD substrates that are degraded by a previously unknown PQC mechanism.

**Figure 3:**
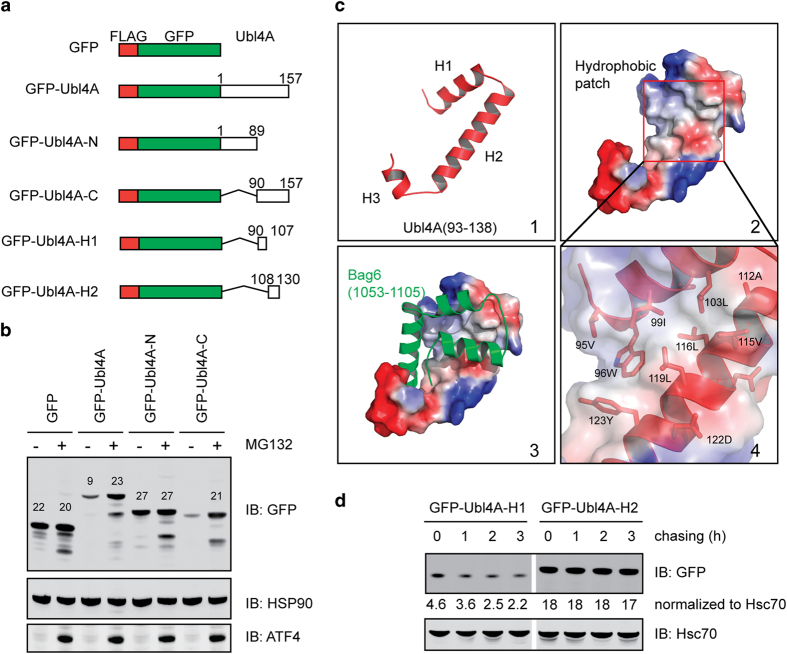
An exposed hydrophobic residue-containing segment destabilizes unassembled Ubl4A. (**a**) A schematic illustration of the Ubl4A constructs used in the study. (**b**) The C-terminus of Ubl4A contains a ‘degron’. HEK293T cells transfected with the indicated plasmids were treated with dimethyl sulfoxide (−) or 10 μm MG132 (+) for 20 h. Whole-cell extracts were analyzed by immunoblotting. (**c**) Structural analysis of the Ubl4A-Bag6 interactions. Panel 1, The C-terminus of Ubl4A contains 3 helices (H1, H2 and H3). Panel 2, A surface view of the Ubl4A C-terminus. Residues are colored by hydrophobicity. Panel 3, Bag6 shields the hydrophobic residues in H1 and H2. Panel 4 shows a close-up view of the boxed area in panel 2. (**d**) Cycloheximide chase analysis of cells transfected with GFP appended with either the H1 or H2 segment from Ubl4A.

To better define this new PQC pathway, we characterized the destabilizing signal in unassembled Ubl4A. Ubl4A contains a N-terminal ubiquitin-like (UBL) domain that binds the Bag6 co-chaperone SGTA and a C-terminal segment that binds Bag6 [20]. When fused to GFP (Figure 3a), both full-length Ubl4A and the C-terminal Ubl4A fragment converted GFP into an unstable protein whose level could be increased by MG132 treatment (Figure 3b). These results suggest that the destabilizing signal is located in the Ubl4A C-terminus.

Structural studies have shown that the Ubl4A C-terminus contains two regular helices (H1 and H2) and a short half-helix (H3; Figure 3c, panel 1) [20]. H1 and H2 collectively form a concave that interacts with Bag6 (panels 2 and 3). The binding interface, decorated by several hydrophobic residues from H1 and H2 (panel 4), would be exposed in the absence of Bag6, which might cause Ubl4A degradation. To test this idea, we appended either H1 or H2 to the C-terminus of GFP. A translation shut-off experiment showed that appending H1 but not H2 to GFP destabilized it (Figure 3d). Thus, the degradation signal in unassembled Ubl4A is a hydrophobic residue-containing segment normally embedded when Ubl4A forms a complex with Bag6.

### HUWE1 is a ubiquitin ligase for unassembled Ubl4A

**Figure 4:**
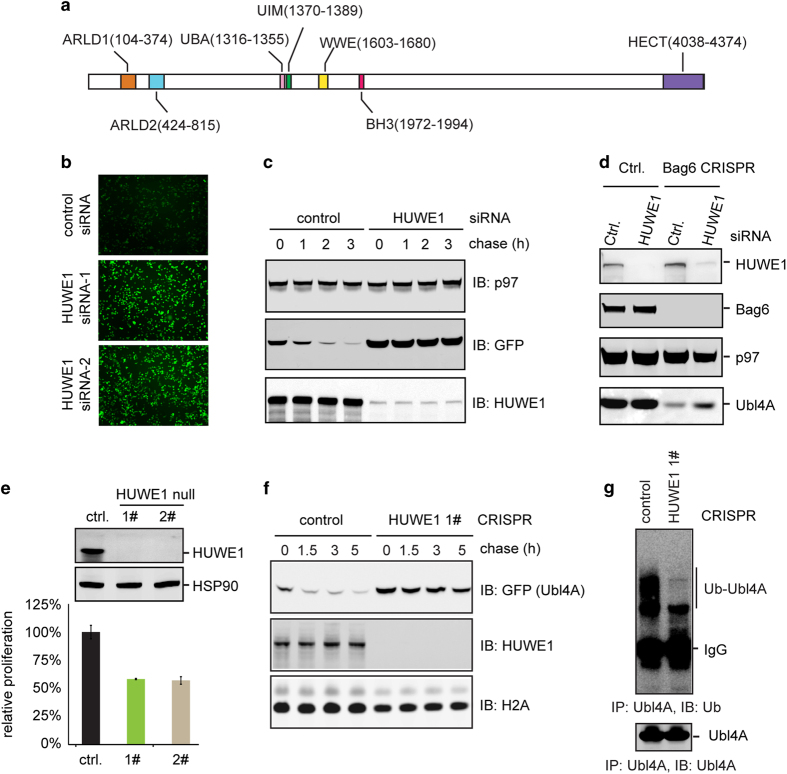
HUWE1 is required for degradation of unassembled Ubl4A. (**a**) The domain structure of HUWE1. BH3, BCL-2 homology; UBA, ubiquitin-associated; UIM, ubiquitin-interacting motif. (**b**, **c**) siRNA-mediated knockdown of HUWE1 stabilized Ubl4A-GFP. (**b**) Bag6 CRISPR cells stably expressing Ubl4A-GFP were transfected with two different HUWE1 siRNAs. Cells were imaged 48 h post transfection. (**c**) Cycloheximide chase analysis of Ubl4A-GFP degradation in Bag6 CRISPR cells stably expressing Ubl4A-GFP. Where indicated, cells were transfected with control or HUWE1 siRNA. (**d**) HUWE1 knockdown stabilizes endogenous Ubl4A in Bag6 null cells. (**e**) Generating HUWE1 null HEK293 T CRISPR cells. Two HUWE1 null clones were obtained. The graph indicates the relative rate of proliferation. Error bars, s.e.m. (*n*=3). (**f**) Cycloheximide chase analysis of Ubl4A-GFP degradation in control and HUWE1 CRISPR cells. (**g**) HUWE1 is required for ubiquitination of unassembled Ubl4A. Ubl4A overexpressed in either control or HUWE1 null cells was analyzed by immunoprecipitation and immunoblotting with ubiquitin (top panel) and Ubl4A (bottom panel) antibodies.

Because unassembled Ubl4A is not degraded by any of the tested PQC ubiquitin ligases (Supplementary Figure S3), we wished to identify the ubiquitin ligase(s) responsible for its instability. As the ‘degron’ on unassembled Ubl4A is featured by hydrophobic residues, we designed an siRNA library targeting a collection of ubiquitin ligases (14) and accessory proteins known to interact with chaperones. As a positive control, we used siRNA that targets p97. A Bag6 null CRISPR cell line stably expressing Ubl4A-GFP was transfected with these siRNAs. Cell extracts prepared 72 h post-transfection were analyzed by immunoblotting. As expected, knockdown of p97 increased Ubl4A-GFP by ~3-fold (Supplementary Figure S4A). Among the other siRNAs analyzed, the only one that caused a consistent accumulation of Ubl4A-GFP targets a large homologous to the E6-AP carboxyl terminus (HECT) domain-containing ubiquitin ligase named HUWE1 (HECT, UBA and WWE domain containing 1, also known as MULE; Supplementary Figure S4A, Figure 4a).

To validate the involvement of HUWE1 in USPD, we synthesized two additional HUWE1 specific siRNAs. A fluorescence imaging experiment confirmed that Ubl4A-GFP in Bag6 null cells was significantly upregulated on knockdown of HUWE1 (Figure 4b). This was apparently due to increased stability because a translation shut-off experiment showed that knockdown of HUWE1 almost completely blocked Ubl4A turnover (Figure 4c). Importantly, immunoblotting confirmed that knockdown of HUWE1 also increased the level of endogenous Ubl4A in Bag6 null cells (Figure 4d), demonstrating that HUWE1 is required for degradation of unassembled Ubl4A of endogenous source.

Because of the potential off-target effect of siRNAs, we used the CRISPR-mediated gene inactivation approach to further validate the role of HUWE1 in USPD. To minimize off-target effect of CAS9, we used the CAS9 D10A nickase-based strategy, which requires simultaneous matching two guiding sgRNAs to a template to inactivate the target gene [28]. Two clones of HEK293T cells lacking HUWE1 were generated (Figure 4e). Compared with control clone, HUWE1 null cells grew more slowly (Figure 4e). A translation shut-off experiment showed that in HUWE1 null cells the degradation of overexpressed Ubl4A was dramatically inhibited (Figure 4f). Furthermore, when we analyzed Ubl4A purified by immunoprecipitation under denaturing conditions by immunoblotting, we found that inactivation of HUWE1 consistently reduced Ubl4A ubiquitination (Figure 4g). Together, these results strongly indicate HUWE1 as a ubiquitin ligase responsible for ubiquitination and degradation of unassembled Ubl4A.

Interestingly, although HUWE1 CRISPR cells had a higher level of FNTA expression than control cells, the degradation kinetics of FNTA was similar between these cells (Supplementary Figure S4B). Importantly, depletion of HUWE1 did reduce ubiquitinated FNTA (Supplementary Figure S4C). These results support the notion that the UBR family and HUWE1 each mediate the degradation of a distinct class of USPD substrates, represented by FNTA and Ubl4A, respectively.

### Identification of endogenous HUWE1 substrates

**Figure 5:**
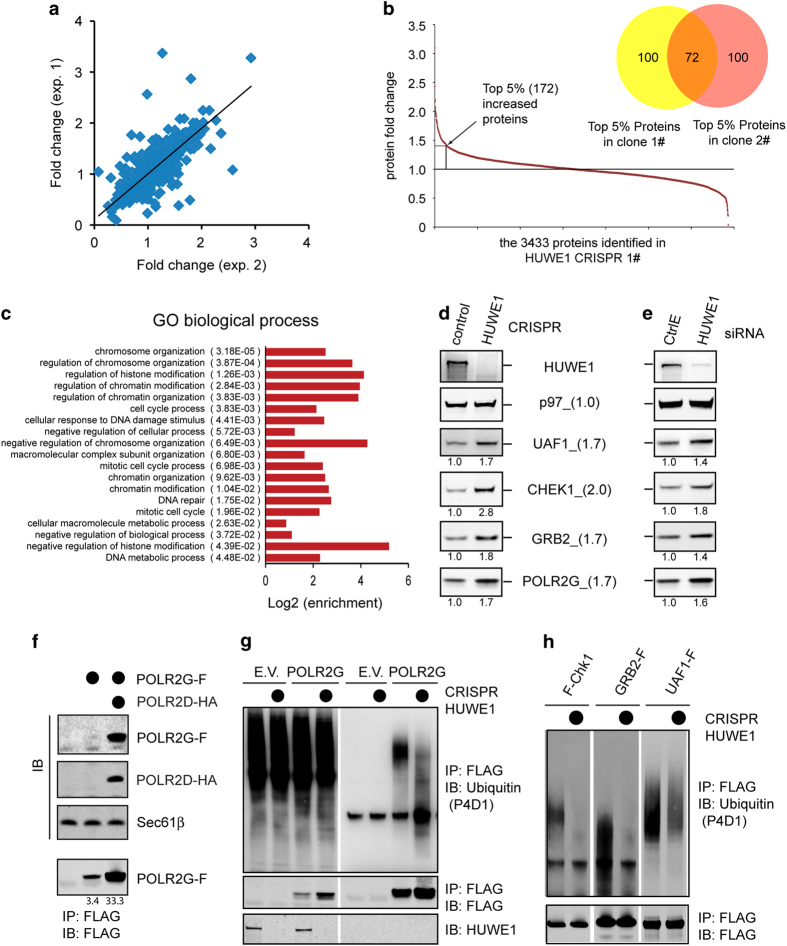
Identification of endogenous HUWE1 substrates. (**a**) Protein fold change comparison between two independent SILAC experiments (exp.). (**b**) The range of protein fold changes for the 3 433 proteins identified in HUWE1 knockout clone 1. The Venn diagram compared the top 172 proteins increased upon HUWE1 inactivation in the two different CRISPR clones. (**c**) The biological processes affected by HUWE1 inactivation. (**d**) Immunoblotting analyses validate the stabilization of proteins involved in DNA damage response pathways using HUWE1 CRISPR cells. The numbers in parentheses indicate fold changes measured by SILAC. The numbers under each gel panel show the band intensity. (**e**) Immunoblotting validation using HEK293T cells transfected with control or HUWE1 siRNA. (**f**) Unassembled POLR2G is unstable. Immunoblotting analysis of cells transfected with FLAG-tagged POLR2G (POLR2G-F) either alone or together with HA-tagged POLR2D. (**g**, **h**) The ubiquitination state of substrates in control and HUWE1 CRISPR cells. (**g**) Control or HUWE1 CRISPR cells were transfected with either an empty vector (EV) or a construct expressing FLAG-tagged POLR2G. Cell lysate were subject to immunoprecipitation under denaturing conditions and analyzed by immunoblotting. (**h**) Cell lysates from control or HUWE1 CRISPR cells transfected with the indicated HUWE1 substrates were subject to immunoprecipitation using FLAG beads. Precipitated proteins were analyzed by immunoblotting.

To elucidate the physiological relevance of HUWE1-mediated PQC, we used stable isotope labeling with amino acids (SILAC)-based quantitative mass spectrometry to identify endogenous HUWE1 substrates. To this end, control and two HUWE1 null CRISPR cell lines were grown in medium supplemented with light or heavy arginine residues. Cell extracts prepared from a mixture of control and HUWE1 null cells were subject to mass spectrometry analyses to determine the ratio of heavy to light amino-acid-labeled proteins. A comparison of data from three biological repeats showed that the study was highly reproducible (Figure 5a, data not shown). Collectively, these experiments identified a total of 3433 proteins in both HUWE1 null clones. For the vast majority of the identified proteins, the ratio between heavy and light arginine-labeled peptides was close to 1, suggesting that the abundance of the corresponding proteins was comparable. However, ~5% of the proteins (172) had a heavy to light arginine ratio above 1.36 or 1.43 in clones 1 and 2, respectively, indicative of increased protein abundance in HUWE1 null cells compared with control cells (Supplementary Table S1). Among them, 72 proteins (42%) were identified in both HUWE1 null clones (Figure 5b). This group contains MCL1, a previously established HUWE1 substrate [29] (Protein fold change: 1.4 and 1.7 in clones 1 and 2, respectively), suggesting that these proteins are probably endogenous HUWE1 substrates.

According to the UniProt database, the identified putative HUWE1 substrates consist of 26 nuclear proteins and 18 cytosolic proteins. The remaining polypeptides are present in both compartments (Supplementary Figure S5A). 88% (63) of the identified proteins are known to function in multi-protein complexes (Supplementary Table S2). Gene ontology (GO) annotation suggests that many of these proteins are involved in nucleic acid metabolism or cell cycle regulation (Figure 5c). For example, the list is enriched in proteins involved in DNA damage response pathways (CHEK1, GRB2, INO80E, LIG1, MCL1, UBE2B, PRKDC, POLR2G, UAF1, RECQL, UBE2E2, TRIP12 and MORF4L1). We used immunoblotting to confirm that at the endogenous level, several proteins in this pathway such as CHEK1, GRB2, POLR2G and UAF1 were indeed accumulated to higher levels on HUWE1 depletion (Figure 5d and e). The increase, albeit modest, was highly reproducible, and was observed in both HUWE1 knockdown and CRISPR null cells. Furthermore, the protein fold changes measured by immunoblotting were also consistent with mass spectrometry analyses (Figure 5d and e). The stabilization of these proteins on HUWE1 inactivation was further confirmed by translation shut-off analyses (Supplementary Figure S5B and C).

Among the proteins examined, POLR2G is known to associate with POLR2D, forming a sub-complex that is further assembled into a large Pol II RNA polymerase complex [30]. Structural analysis showed that POLR2G has a large surface featured by hydrophobic amino acids, which are shielded when it associates with POLR2D (Supplementary Figure S5D) [31]. Immunoblotting showed that similar to Ubl4A, POLR2G could not be expressed at high levels unless its partner POLR2D was present (Figure 5f). Furthermore, its level could be enhanced by treatment with MG132 or NMS-873 (Supplementary Figure S5E). Co-immunoprecipitation showed that like Ubl4A, overexpressed POLR2G also associated with endogenous HUWE1 (Supplementary Figure S5F). These results suggested that at least some of the identified proteins are genuine HUWE1 substrates degraded due to inefficient assembly: the modest accumulation of these substrates in HUWE1 null cells presumably reflects the fact that cells have evolved to produce functional complexes and thus only a small fraction of the proteins are subject to USPD at any given time.

To test whether the identified substrates undergo HUWE1-dependent ubiquitination in cells, we overexpressed these proteins bearing a C-terminal FLAG tag in HEK293T cells. Immuoprecipitation under denaturing conditions demonstrated that these proteins were all ubiquitinated in control cells even without inhibition of the proteasome (Figure 5g and h, Supplementary Figure S5G). In HUWE1 null cells, ubiquitinated CHK1, MORF4L1 and GRB2 were almost undetectable, whereas UAF1 and SNX17 ubiquitination was reproducibly reduced (Figure 5h, Supplementary Figure S5C). For POLR2G, it contained shortened ubiquitin chains in HUWE1 null cells compared with that from control cells, suggesting that it is inefficiently ubiquitinated in the absence of HUWE1 (Figure 5g, Supplementary Figure S5G). The only protein examined that did not show reduced ubiquitination was NOC2L. NOC2L is probably not a HUWE1 substrate and its accumulation in HUWE1 null cells is likely due to secondary effects, but the other proteins analyzed are clearly subject to HUWE1-dependent ubiquitination. Our data also suggest that certain substrates such as POLR2G may employ more than one ubiquitin ligase for ubiquitination.

### HUWE1 degrades unassembled proteins in the cytoplasm

GO database annotations indicate that many HUWE1 substrates function in the nucleus. To validate this conclusion, we used antibodies to stain cells transiently expressing several identified putative HUWE1 substrates. Indeed, among 8 proteins examined, all but one (UAF1) was detected predominantly in the nucleus, co-localizing with DAPI staining (Supplementary Figure S6, data not shown).

**Figure 6:**
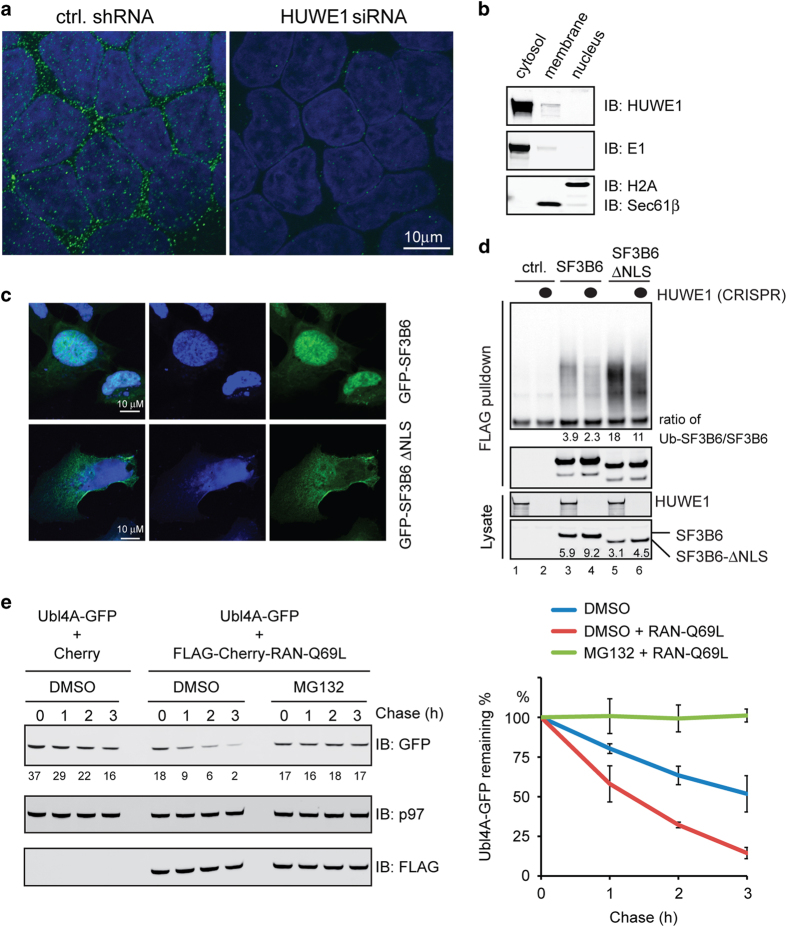
HUWE1 acts in the cytoplasm in USPD. (**a**) Localization of HUWE1 by immunostaining of cells transfected with either control or HUWE1 siRNA. (**b**) Biochemical fractionation analysis of HUWE1 localization in HEK293T cells. (**c**) Removal of NLS alters the localization of SF3B6. HeLa cells transfected with SF3B6-GFP or SF3B6∆NLS-GFP were stained with DAPI and imaged. (**d**) Deletion of the NLS increases SF3B6 ubiquitination by HUWE1. Lysates from control or HUWE1 CRISPR cells transfected as indicated were subject to immunoprecipitation under denaturing conditions with anti-FLAG beads. Immunoprecipitated proteins were analyzed by immunoblotting. (**e**) Expression of RAN Q69L inhibits degradation of unassembled Ubl4A. 293T Cells transfected as indicated were treated with cycloheximide in the presence of dimethyl sulfoxide as a control or MG132 (20 μM). Cell lysates prepared at the indicated time points were analyzed by immunoblotting. The graph shows the quantification of three experiments. Error bars, s.e.m. (*n*=3).

We next used immunostaining to examine the subcellular localization of HUWE1 in HEK293T cells. Interestingly, although HUWE1 substrates were predominantly localized in the nucleus, HUWE1 itself was localized in small punctae distributed throughout the cytoplasm (Figure 6a). Both the number and the intensity of HUWE1 positive punctae were reduced in cells transfected with a HUWE1 siRNA, demonstrating the antibody specificity (Figure 6a). The cytoplasmic localization of HUWE1 was further confirmed by a biochemical fractionation experiment (Figure 6b).

The spatial segregation of HUWE1 from its substrates suggested two possible mechanisms by which HUWE1 can target a USPD substrate. First, an unassembled USPD protein may be exported out of the nucleus before ubiquitination by HUWE1. Alternatively, HUWE1 may act in the cytosol during the assembly process to degrade polypeptides that are not efficiently assembled. The second model is conceptually analogous to ERAD-dependent PQC, which operates concurrently with membrane protein biogenesis.

To distinguish between these possibilities, we used two approaches to inhibit nuclear transport of HUWE1 substrates. If degradation occurs before nuclear import, inhibition of nuclear transport should cause more substrates to be degraded by HUWE1. First, we deleted the predicted nuclear localization signal (NLS) of SF3B6, a protein upregulated in HUWE1 null cells (Supplementary Figure S5A). As expected, this resulted in the re-localization of SF3B6 to the cytoplasm (Figure 6c). Immunoprecipitation showed more SF3B6 ∆NLS in ubiquitinated forms than the WT counterpart despite its reduced steady state level (Figure 6d, lanes 5 vs 3). This was at least in part due to HUWE1-dependent ubiquitination because like WT SF3B6, the level of ubiquitinated SF3B6 ∆NLS was reduced in HUWE1 null cells (lanes 4 and 6 vs 3 and 5). Thus, inhibition of nuclear transport of SF3B6 by removing the NLS increases HUWE1-dependent ubiquitination. Second, we co-expressed with Ubl4A a mutant RAN GTPase (Q69L) that was reported to inhibit nuclear import [32]. Expression of RAN Q69L reduced the steady state Ubl4A level as a result of increased turnover by the proteasome (Figure 6e). These results, together with the cytosolic localization of HUWE1, strongly suggest that ubiquitination and degradation of unassembled nuclear USPD substrates by HUWE1 occurs in the cytoplasm, before their entry into the nucleus.

## Discussion

In this study, we establish model substrates to study the mechanism of USPD. We discover a PQC pathway that uses HUWE1 as the ubiquitin ligase to target unassembled soluble proteins for proteasomal degradation. HUWE1 appears to act in parallel with the UBR1 ubiquitin ligase family: while UBR1 and the related enzymes primarily target misfolded or unassembled proteins bearing an exposed N-terminal destabilizing amino acid, the degradation signal for HUWE1 substrates is exposed segment containing hydrophobic residues. Our findings are consistent with a recent report, which demonstrated that in *S. cerevisiae* the HUWE1 homolog Tom1 can facilitate the degradation of unassembled ribosome subunits bearing exposed hydrophobic surface [33]. Together, these findings underscore the biological importance of an evolutionarily conserved PQC mechanism that targets unassembled soluble proteins for degradation.

Our study shows that both N-end rule- and HUWE1-mediated USPD require the ubiquitin selective chaperone p97 and the co-factor Npl4. However, unlike the UBR1 family, HUWE1 is a giant (450 kD) ubiquitin ligase, and in addition to the HECT ubiquitin ligase domain, it contains many protein-protein interaction motifs. Among the reported interactions, it is noteworthy that HUWE1 was identified as a component of the Hsp27 interactome [34]. This provides a possible explanation for the hydrophobic nature of the identified ‘degron’ on HUWE1 substrates. In addition to unassembled proteins, HUWE1 also targets certain folded polypeptides for ubiquitination [35–37]. Given the substrate diversity, HUWE1 likely binds additional chaperones or substrate recruiting adapters. A thorough characterization of the HUWE1 interactome will be needed to elucidate the mechanism of substrate recognition in HUWE1-mediated degradation.

**Figure 7:**
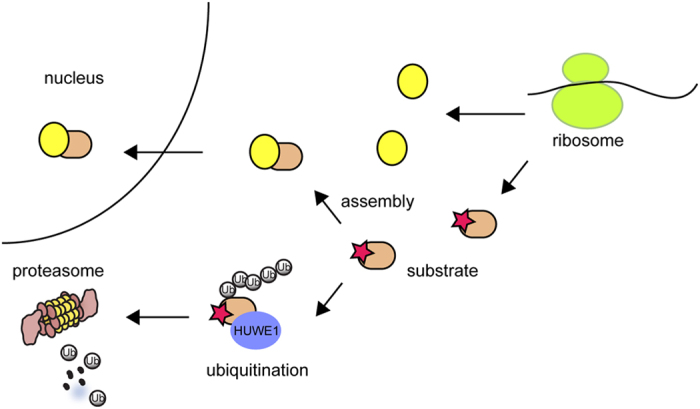
A model of HUWE1-mediated cytosolic PQC. On translation, proteins are assembled into multi-subunit protein complexes. The assembly process usually shields hydrophobic surfaces that would otherwise be exposed on unassembled subunit. Although assembled protein complexes can be transported to their final destination (for example, nucleus), unassembled protein subunits bearing an exposed hydrophobic segment (marked by the star) is subject to HUWE1-mediated ubiquitination, which targets them for degradation by the proteasome in the cytosol.

Protein assembly is an essential step in protein biogenesis. The assembly process can be quite complex, particularly for large assemblies. How cells control protein assembly to ensure correct stoichiometry of the final product is unclear. Unlike prokaryotes that use operons to synchronize the expression of proteins involved in the same process, subunits of a given protein complex in eukaryotic cells are not produced in a coordinated manner. Therefore, unassembled proteins are unavoidable, and their removal is required to maintain the correct stoichiometry of protein assemblies in eukaryotic cells. To maximize the assembly efficiency, protein assembly may take place in certain subcellular locations, coupled to protein translation as in the case of ER protein biogenesis. In the ER, PQC regulators are often located in proximity to the assembly machinery. In fact, many ER chaperones involved in protein folding and assembly also function in the triaging process that targets terminally misfolded proteins for degradation. Conceptually, this would allow the assembly process to be closely monitored for ‘defective pieces’. Notably, HUWE1 is localized to small foci throughout the cytoplasm, which might represent a site of protein assembly factory. Interestingly, our study shows that many HUWE1 substrates are components of large nuclear protein assemblies. Constitutive degradation of a small fraction of these nuclear proteins by HUWE1 is an indication of incomplete assembly at a low basal level, suggesting the existence of an embedded PQC mechanism that balances nuclear protein assembly and turnover. Thus, like ER protein biogenesis, nuclear protein assembly, which takes place in the cytosol, is subject to surveillance by a cytosolic PQC pathway orchestrated by HUWE1 (Figure 7).

Unassembled proteins are detrimental not just because they cannot execute a desired function, more often it is due to a dominant negative effect on essential cell physiology. This could be caused by either protein aggregation or non-specific interactions. Alternatively, incompletely assembled protein complexes could retain partial function. Consequently, they could interfere with the normal function of a correctly assembled complex. This appears particularly precarious for nuclear proteins, which often function in large complexes with each subunit executing a specific function. For instance, a transcription factor complex missing a subunit bearing a transactivation domain can obviously bind DNA to inhibit rather than activate transcription. Therefore, unassembled nuclear proteins pose no lesser a threat to the protein homeostasis network than misfolded or misassembled cytosolic or membrane proteins. In this regard, HUWE1-mediated PQC may influence diverse cellular processes, as suggested by recent studies [35–37].

It is noteworthy that a recent proteomic study using a breast gland-derived cell line to interrogate the impact of HUW1 depletion on the cellular ‘ubiquitome’ reveals a list of putative HUWE1 substrates that show little overlapping with the proteins identified in this study [38]. The discrepancy is probably caused by the different tissues (293 T vs BT-549) used in these studies as it is conceivable that the assembly efficiency for protein complexes may vary between different tissues. In addition, it is also possible that some identified HUWE1 substrates may be normal proteins degraded in a tissue specific manner for a regulatory reason. Indeed, a recent report showed that in quiescent cells, HUWE1 can target histone H2AX for degradation, but this process is blocked by DNA damage as DNA damage can cause relocation of HUWE1 to DNA damage foci in the nucleus [36, 37]. These findings further underscore the importance of the cytosol-localized HUWE1 in degradation of nuclear proteins, and also suggest that HUWE1-mediated degradation could be subject to regulation by environmental stimuli.

## Materials and methods

### Cell lines, plasmids and siRNAs

The HEK293T cell was purchased from ATCC. The cells were maintained in Dulbecco's modified eagle medium (DMEM) medium (Corning cellgro) containing 10% fetal bovine serum and penicillin-streptomycin (10 units per ml). Mammalian expression constructs for FLAG-Bag6, UAF1-FLAG, RGS-His-p97 WT and RGS-His-p97-QQ mutant were described previously [23, 39, 40]. The plasmid expressing Bag6-His was a gift from Dr Ramanujan Hegde. FLAG-Bag6-∆C81 was a gift from Dr Bil Clemons lab (California Institute of Technology). The plasmids expressing Ubl4A-FLAG, POLR2G-FLAG, SRP14-FLAG, SRP14- FLAG, SNX17-FLAG and NOC2L-FLAG were purchased from Origene. pmCherry-C1-RAN-Q69L was a gift from Jay Brenman (Addgene plasmid # 30309). The mammalian expression constructs for Ubl4A-GFP were made by insert Ubl4A open reading frame between SacII and BamHI sites of pVENUS-N1 vector. To express FLAG-GFP-Ubl4A, FLAG-GFP-Ubl4A-N(1-89), FLAG-GFP-Ubl4A-C(90-157), FLAG-GFP-Ubl4A-H1(90-107), FLAG-GFP-Ubl4A-H2(108-130), we first inserted a FLAG-encoding oligonucleotide at the 5ʹ of GFP gene in pEGFP-C1 vector without changing the multiple cloning site of the vector. Next, Ubl4A full-length complementary DNA (cDNA) or cDNA segments indicated in the Figure 3a were amplified by PCR and inserted into the plasmid. To construct FNTA-FLAG, FNTA-GFP, FNTB-HA, GFP-SF3B6-FLAG, GFP-SF3B6-∆NLS-FLAG, POLR2G-GFP, GRB2-FLAG, FLAG-MORF4L1(isoform2) and FLAG-CHK1, the open reading frame of these genes amplified from a HEK293T cDNA library were inserted into mammalian expression vectors. pX330-U6-Chimeric_BB-CBh-hSpCas9 was a gift from F. Zhang (Addgene 42230). The D10A mutation in CAS9 was introduced by site-directed mutagenesis.

To generate CRISPR knockout cell lines, two CAS9 D10A nickase-based constructs targeting each gene were designed according to the published protocol [28]. In brief, oligonucleotides targeting HUWE1 are:

Target 1, forward primer: 5ʹ-caccgatacacctactccccgattc-3ʹ

Target 1, reverse primer: 5ʹ-aaacgaatcggggagtaggtgtatc-3ʹ

Target 2, forward primer: 5ʹ-caccgaggctgttgatcgcgcggc-3ʹ

Target 2, reverse primer: 5ʹ-aaacgccgcgcgatcaacagcctc-3ʹ

Oligonucleotides targeting Bag6 are:

Target 1, forward primer: 5ʹ-caccggaggtgttggtgaagacct-3ʹ

Target 1, reverse primer: 5ʹ-aaacaggtcttcaccaacacctcc-3ʹ

Target 2, forward primer: 5ʹ-caccgaggctcctccacagcggtac-3ʹ

Target 2, reverse primer: 5ʹ-aaacgtaccgctgtggaggagcctc-3ʹ

Each pair of oligonucleotides (10 μm) in distilled water were heated at 95 °C for 5 min and annealed by ramping down the temperature from 95 °C to 25 °C at 5 °C per minute. The annealed oligonucleotides were ligated into pX330-hSpCas9 containing the D10A mutation using the BbsI ligation sites. The two HUWE1 targeting constructs were co-transfected into HEK293T cells. 24 h post-transfection, cells were diluted and seeded into 96-well plate at <1 cell per well. Clones derived from single cells were obtained and screened for HUWE1 or Bag6 deficiency by immunoblotting. Transfection was performed with TransIT-293 (Mirus) for HEK293T cells.

To make a stable cell line expressing Ubl4A-GFP, the Ubl4A gene was first inserted into the pVENUS-N1 vector between SacII and BamHI sites. The construct was transfected into Bag6 null CRISPR cells. One the following day, fresh media containing G418 was added for selection. The selection media was replaced every 2 days until most cells were killed and washed off. The remaining cells were sorted by a FACS machine. Cells positive for green fluorescence were pooled and the Ubl4A-GFP expression was confirmed by immunoblotting.

siRNAs for gene knockdown and corresponding control siRNA were listed in Supplementary Table S3. siRNA pool library for screening E3 ligase(s) responsible for Ubl4A-GFP degradation is described in Supplementary Table S4.

### Antibodies, chemicals and proteins

The antibody for HUWE1 was purchased from Bethyl Laboratories, Inc. (Montgomery, TX, USA, Cat. No. A300-486A). GFP, Bag6 and Ubl4A antibodies were described previously [41]. MG132 was purchased from EMD.

### Immunoblotting and immunoprecipitation

Cells were lysed in the NP40 lysis buffer containing 50 mm Tris-HCl pH 7.4, 150 mm sodium chloride, 2 mm magnesium chloride, 0.5% NP40 and a protease inhibitor cocktail. Cell extracts were subject to centrifugation at 17 000 *g* 5 min to remove insoluble materials. To prepare whole-cell extract, the cells were lysed by the Laemmli buffer directly. Immunoblotting was performed according to the standard protocol. Fluorescence-labeled secondary antibodies (Rockland, Rockville, MD, USA) were used for detection. The fluorescent bands were imaged and quantified on a LI-COR Odyssey infrared imager using the software provided by the manufacturer. For immunoprecipitation, the whole-cell extract was incubated with FLAG-agarose beads (Sigma, St Louis, MO, USA) or protein A-Sepharose CL-4B (GE Healthcare, Piscataway, NJ, USA) bound with antibodies against specific proteins. After incubating, the beads were washed two times by the NP40 wash buffer containing 50 mm Tris-HCl pH 7.4, 150 mm sodium chloride, 2 mm magnesium chloride, 0.1% NP40. The proteins on beads were assayed by immunoblotting.

### Immunoprecipitation under denaturing conditions

To detect ubiquitin conjugates on specific proteins, cells (5×10^5^) were seeded and grown for 24 h, and then transfected with specific plasmids or control plasmids (400 ng). Cells were collected 24 h post-transfection, re-suspended in 75 μl PBS. 75 μl buffer D (2% SDS, 5 mm DTT) was added to the cells and the cells were immediately heated at 95 °C for 10 min. Cell extract was diluted fivefolds with the buffer LNP containing a protease inhibitor cocktail. After centrifugation at 17 000 *g* for 10 min to remove insoluble materials, cleared cell lysates were subject to immunoprecipitation with anti-FLAG M2 Affinity Gel (Sigma-Aldrich, Inc., Cat. No. A2220).

### Quantitative mass spectrometry analysis by SILAC

To compare the protein levels between control CRISPR and HUWE1 null cells, a SILAC (SILAC in cell culture) method was adopted from a previously published report [42]. In brief, cells were maintained for 1 week in heavy medium and light medium for control cell and HUWE1 null cells, respectively. During this process, cells were passaged two times at 1:10 dilution. Once the cells reached confluence, the cells were collected and washed with 10 ml phosphate-buffered saline (PBS). The cells were re-suspended in 1 ml PBS and the cell numbers were counted. A total of 4.5 million control and HUWE1 null cells were mixed. The supernatant was removed by centrifugation and the cell pellets were treated with 0.5 ml NP40 lysis buffer (50 mm Tris-HCl pH 7.5, 0.5% NP40, 5 mm magnesium chloride, and 150 mm sodium chloride, 1 mm DTT) containing a protease inhibitor cocktail on ice for 15 min. The lysates were subject to centrifugation at 17 000 *g* for 10 min. The protein concentrations in the supernatant fractions were determined by Bio-Rad Protein Assay Dye Reagent (Cat. No. 500-0006). The protein concentration was adjusted to 4.8 mg ml^−1^ by addition of NP40 lysis buffer. For each mass spectrometry analysis, 0.4 ml of lysate was mixed with 3.2 ml ice-cold acetone. After mixing, we added 0.4 ml 100% trichloroacetic acid (TCA) to the solution to precipitate the proteins. After incubation at −20 °C for 1 h, precipitated proteins were spun down at 17 000 *g* for 10 min. The pellet was washed with 1 ml ice-cold acetone and spin down at 17 000 *g* for 10 min. After removal of the supernatant, the pellet was stored at −80 °C until mass spectrometry analysis.

For protein mass spectrometry analysis, samples were denatured in urea, reduced and alkylated, digested down to tryptic peptides, desalted by reversed phase, separated into six fractions using cation exchange, data collected using long gradient LC/MS/MS, and data analyzed using MaxQuant.

### Protein stability analysis and biochemical fractionation

To conduct translation shut-off (cycloheximide chase) experiments, cells were re-suspended in 1.8 ml fresh DMEM containing 50 mm Hepes buffer (pH 7.5) and 50 μg ml^−1^ cycloheximide. Cells were seeded in a 12-well plate with equal number of cells in each well and incubated at 37 °C. At the indicated time points, cells were harvested for immunoblotting analysis.

To obtain different cellular fractionations, HEK293T washed with ice-cold PBS were treated with 5 pellet volume of buffer A (10 mm Tris-HCl pH 7.5, 10 mm potassium chloride, 2 mm magnesium chloride, 0.5 mm DTT) containing a protease inhibitor cocktail on ice for 10 min. The cells were collected by centrifugation at 1 000 *g* for 5 min and re-suspended in 250 μl buffer A before being homogenized by a dounce homogenizer. Homogenized cells were subjected to centrifugation at 600 *g* for 10 min to collect nuclei. The nuclei were washed one more time with buffer A and re-suspended in the Laemmli buffer. The supernatant fractions were further centrifuged at 20 000 *g* to obtain a membrane and cytosol fraction.

### Immunofluorescence experiments

To detect the subcellular localization of protein by fluorescence labeling, cells were seeded on fibronectin-coated cover glass and transfected. Cells were then fixed with phosphate buffer saline containing 2% paraformaldehyde for 20 min at room temperature. Cells were then permeabilized with phosphate buffer saline containing 10% fetal bovine serum and 0.2% saponin, and stained with antibodies in the same buffer according to a standard protocol. Images were acquired with a Zeiss LSM780 confocal microscope or a Zeiss Axiovert 200 inverted microscope.
