# Supplementary material for: The HECT domain ubiquitin ligase HUWE1 targets unassembled soluble proteins for degradation
Source: Cell Discov. 2016 Nov 8;2:16040–. doi: 10.1038/celldisc.2016.40 (PMC5102030; doi:10.1038/celldisc.2016.40)
Supplement: Supplementary Information [file celldisc201640-s1.pdf]

Supplementary Materials

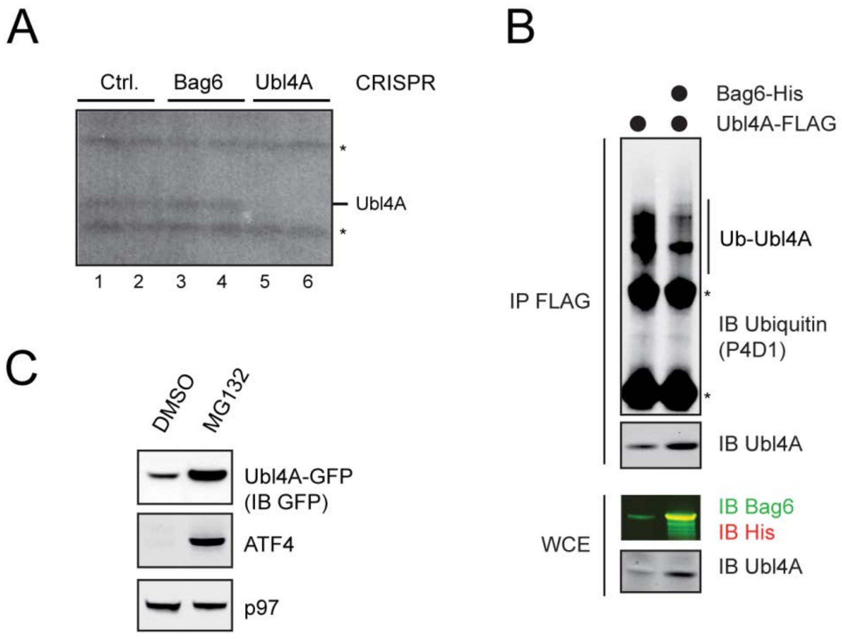

Supplementary Figure S1 Regulation of Ubl4A stability by its assembly partner

A, The translation rate of Ubl4A is not affected in the absence of Bag6. Control, Bag6 and Ubl4A CRISPR cells were radiolabeled with S^{35}-methionine for 5min. Ubl4A immunoprecipitated from cell extracts was analyzed by SDS-PAGE and autoradiography. Asterisks, non-specific bands. **B**, Regulation of Ubl4A ubiquitination by assembly. Bag6 null cells were transfected as indicated. Ubl4A-FLAG immunoprecipitated under denaturing conditions was analyzed by immunoblotting. Asterisks, IgG. **C**, Stabilization of Ubl4A by a proteasome inhibitor. Bag6 CRISPR cells stably expressing Ubl4A-GFP were treated with either DMSO or MG132. Cell lysates were analyzed by immunoblotting with antibodies against the indicated proteins.

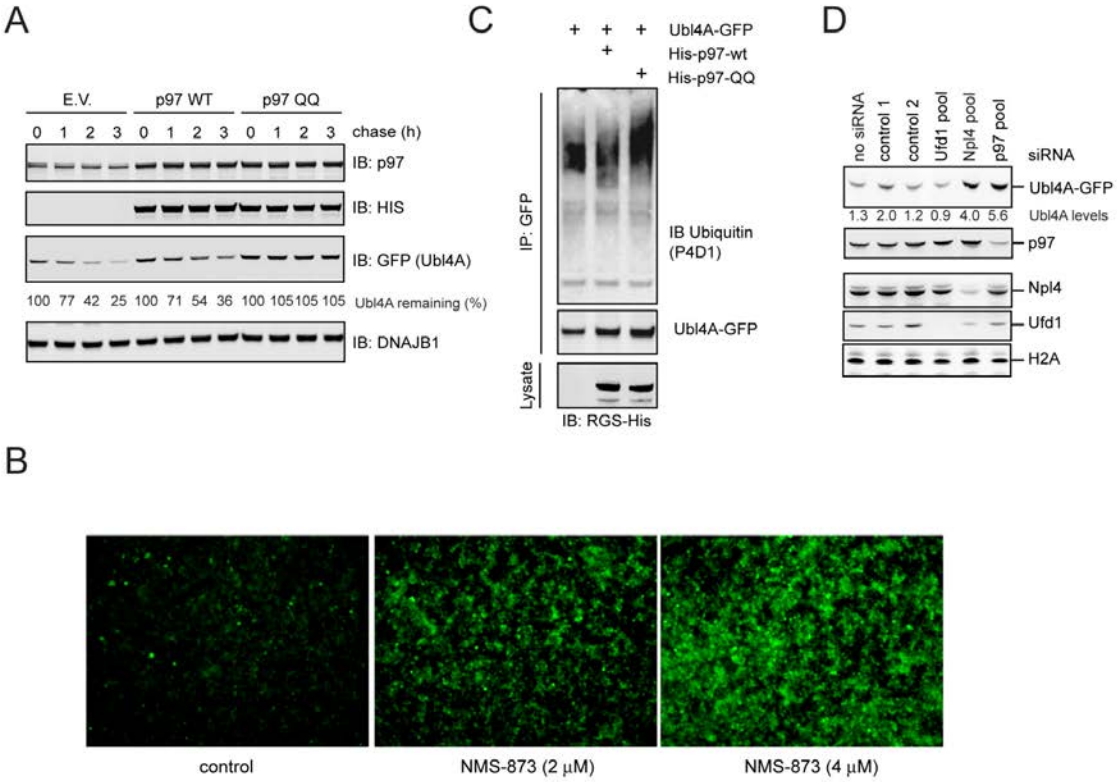

Supplementary Figure S2 Degradation of unassembled Ubl4A requires p97 and Npl4

A, p97 QQ inhibits the degradation of unassembled Ubl4A. HEK293T cells transfected with Ubl4A-GFP together with either empty vector (E.V.), WT p97 or p97 QQ were subject to cycloheximide chase analysis. **B,** Inhibition of p97 by NMS-873 stabilizes Ubl4A-GFP. A stable cell line overexpressing Ubl4A-GFP was treated as indicated for 4.5 h. Cells were imaged using the same exposure time. **C,** p97 QQ expression does not abolish Ubl4A ubiquitination. Ubl4A immunoprecipitated from cells as indicated was analyzed by immunoblotting. **D**, Npl4 knockdown stabilizes unassembled Ubl4A. Bag6 null cells stably expressing Ubl4A-GFP were transfected with the indicated siRNAs. Whole cell extracts were analyzed by immunoblotting with antibodies against the indicated proteins.

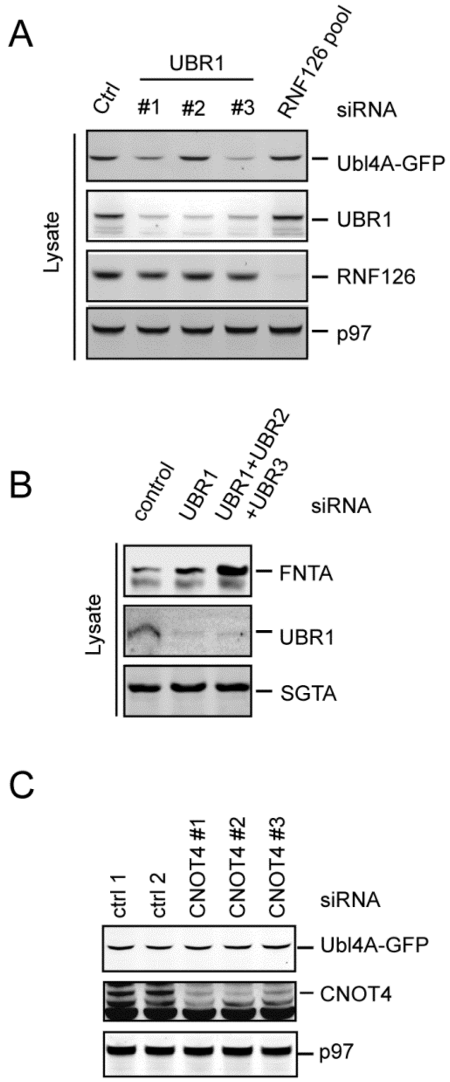

Supplementary Figure S3 Degradation of Ubl4A is not dependent on UBR1 or CNOT4

A, B, The UBR1 family ligases are not required for Ubl4A degradation, but is involved in FNTA degradation. HEK293T cells stably expressing either Ubl4A-GFP **(A)** or transiently expressing FNTA-FLAG **(B)** were transfected with the indicated siRNAs. Whole cell extracts (WCE) were analyzed by immunoblotting. **C**, Knockdown of CNOT4 has no effect on the Ubl4A protein level. As in **A**, except that the indicated siRNAs were used.

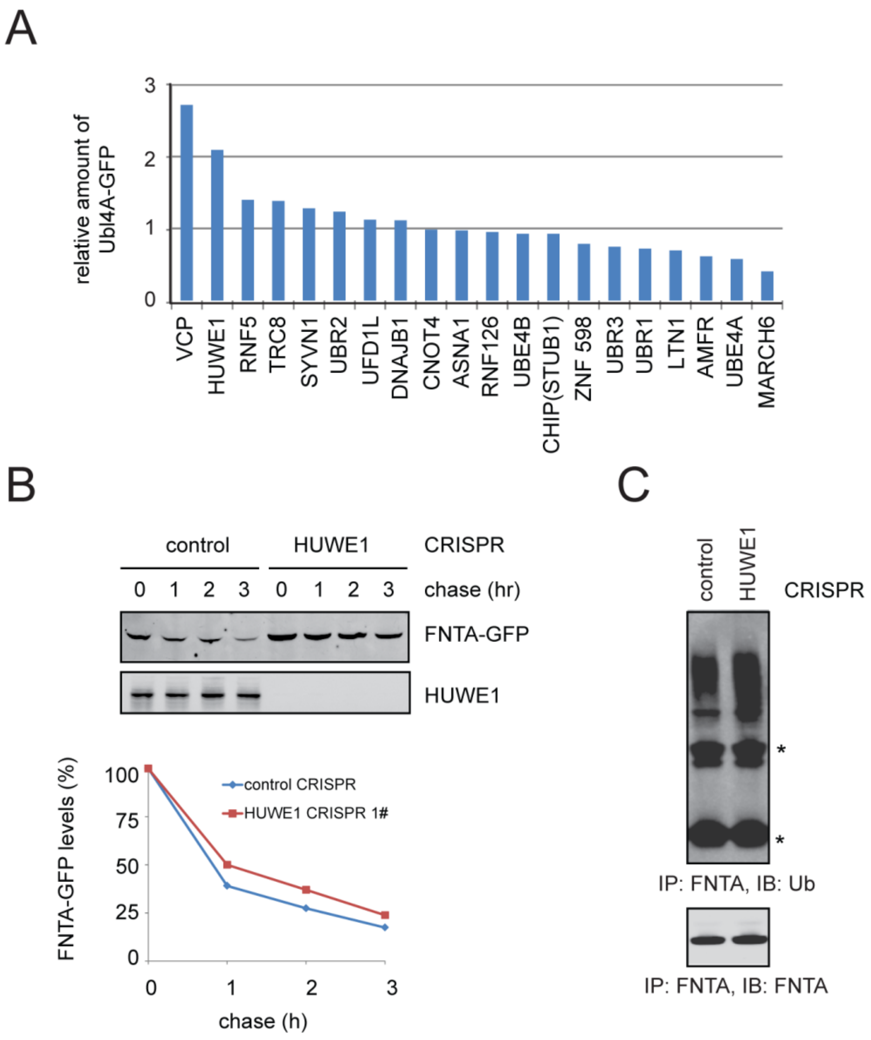

Supplementary Figure S4 HUWE1 is required for Ubl4A degradation, but not for FNTA degradation

A, A siRNA screen to identify ubiquitin ligase involved in Ubl4A degradation. HEK293T cells stably expressing Ubl4A-GFP was transfected with the indicated siRNAs. The Ubl4A protein level in whole cell extract was determined by immunoblotting and plotted relative to the Ubl4A level in cells treated with a VCP (p97) siRNA pool. **B, C,** HUWE1 inactivation does not significantly affect FNTA ubiquitination and degradation. **B,** Translation shut-off analysis of overexpressed FNTA-GFP in control and HUWE1 null cells. The graph indicates the quantification of the gel. **C,** FNTA immunoprecipitated from control and HUWE1 CRISPR cells under denaturing conditions were analyzed by immunoblotting. Asterisks, IgG.

A

Nuc.		Cyt. And Nuc.		Cyt.	
Gene	fold	Gene	fold	Gene	fold
TRIP12	2.03 (±0.14)	CHEK1	1.97 (±0.57)	PLEC	2.19 (±0.05)
NOC2L	2.01 (±0.29)	TTF2	1.91 (±0.30)	DYNLT1	1.97 (±0.12)
SCNM1	1.96 (±0.10)	UBE2B	1.84 (±0.21)	CDC123	1.95 (±0.05)
EIF1AD	1.92 (±0.05)	DNAJB6	1.81 (±0.08)	SNX17	1.81 (±0.06)
SF3B6	1.82 (±0.01*)	NUSAP1	1.78 (±0.17)	CKAP5	1.75 (±0.04)
LIG1	1.79 (±0.18)	RYBP	1.75 (±0.06)	SQLE	1.70 (±0.19)
TAF7	1.72 (±0.03*)	WDR48	1.72 (±0.03)	MOB1A	1.65 (±0.13)
POLR2G	1.71 (±0.07)	PPP2R2A	1.71 (±0.06)	MOB1B	1.65 (±0.13)
MORF4L1	1.63 (±0.02)	GRB2	1.68 (±0.11)	HMGCS1	1.58 (±0.03)
SRSF9	1.57 (±0.06)	CDC34	1.67 (±0.08)	AMD1	1.54 (±0.15)
GRWD1	1.56 (±0.02)	ABCF1	1.57 (±0.01)	VPS53	1.49 (±0.26)
UBE2E1	1.56 (±0.03)	AKAP8L	1.49 (±0.15)	TACC3	1.46 (±0.06)
TBL1XR1	1.54 (±0.06)	ZNF622	1.48 (±0.04)	VPS4B	1.46 (±0.05)
PRKDC	1.52 (±0.00)	GPN1	1.48 (±0.20)	UBXN1	1.45 (±0.09)
IRF2BPL	1.52 (±0.77)	RBM42	1.47 (±0.02)	SRP14	1.44 (±0.02)
WDR82	1.51 (±0.06)	SMAD4	1.40 (±0.11)	GNS	1.43 (±0.13)
ZGPAT	1.50 (±0.08)	MCL1	1.38 (±0.10)	EIF4G3	1.40 (±0.04)
INO80E	1.49 (±0.19)	DAXX	1.38 (±0.17)	CARHSP1	1.38 (±0.06)
FAM60A	1.48 (±0.09)				
BUB1	1.47 (±0.14)				
LEO1	1.46 (±0.09)				
RBBP4	1.46 (±0.02)				
SF1	1.46 (±0.04)				
HNRNPUL2	1.45 (±0.09)				
RECQL	1.39 (±0.03)				
SMARCC2	1.39 (±0.05)				

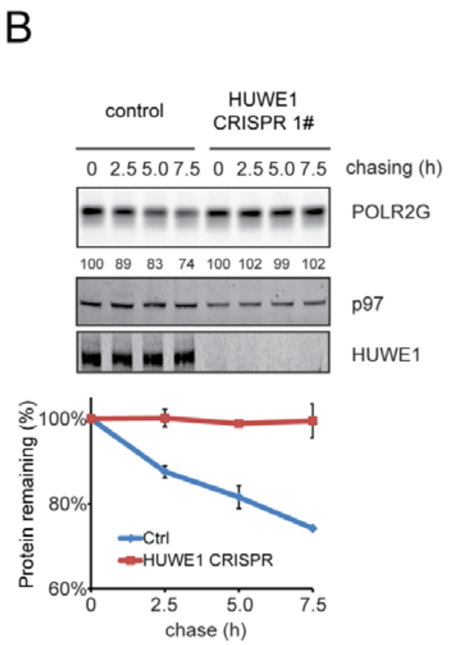

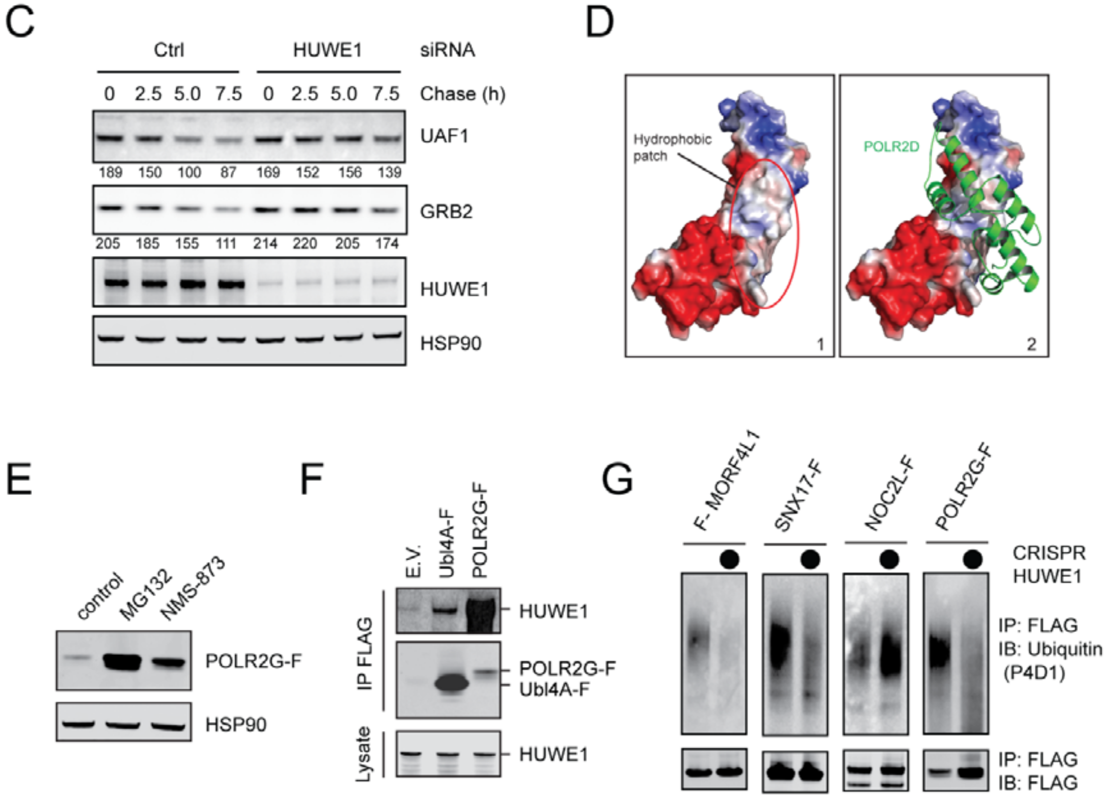

Supplementary Figure S5 Identification of endogenous HUWE1 substrates

A, A list of putative HUWE1 substrates identified by SILAC experiments. The annotated subcellular localizations are indicated. Nuc. Nucleus, Cyt. Cytoplasm. The numbers indicate average fold changes from three independent repeats using HUWE1 CRISPR clone 1. N=3, The numbers in parentheses indicate S.E.M.. Asterisks indicate proteins identified only in two experiments. **B,** Translation shut-off analysis of POLR2G degradation in control and HUWE1 null cells. The error bars of the graph indicate the variation of two independent experiments. **C,** Translation shut-off analysis of the indicated proteins in cells transfected with control or HUWE1 siRNA. **D,** POLR2G has a hydrophobic surface that is shielded by POLR2D. The structural analysis was based on published structure (PDB: 2C35). D, Overexpressed POLR2G can be stabilized by MG132 and NMS-873. **E,** Overexpressed POLR2G can be stabilized by MG132 and NMS-873. **F,** Interaction of Ubl4A and POLR2G with endogenous HUWE1 as analyzed by co-immunoprecipitation. **G,** The ubiquitination status of the indicated proteins was determined by denaturing immunoprecipitation using control and HUWE1 null cells.

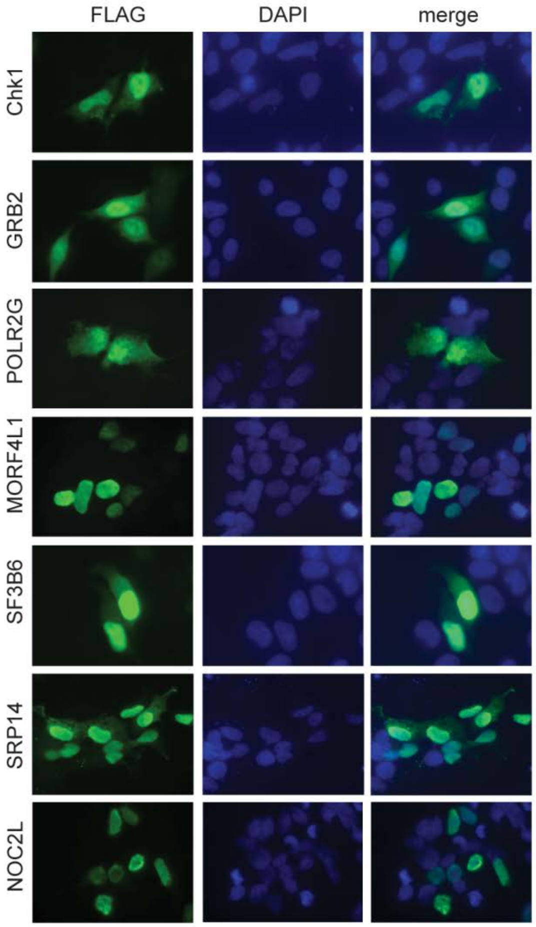

Supplementary Figure S6 The subcellular localization of HUWE1 substrates. HEK293T cells expressing the indicated HUWE1 substrates were stained with FLAG antibodies in green and DAPI in blue.

Supplementary Table 1 A complete list of proteins identified by SILAC analyses in HUWE1null cells clone 1#. Shown is the heavy to light arginine ratio of proteins identified in 3 biological repeats.

Supplementary Table 2 A list of putative HUWE1 substrates with annotated protein complex composition by the Uniprot database.
